# Discussing the Terms Biomimetic and Bioinspired within Bioinorganic Chemistry

**DOI:** 10.1021/acs.inorgchem.4c01070

**Published:** 2024-09-23

**Authors:** Silène Engbers, Phebe H. van Langevelde, Dennis G. H. Hetterscheid, Johannes E. M. N. Klein

**Affiliations:** † Molecular Inorganic Chemistry, Stratingh Institute for Chemistry, Faculty of Science and Engineering, University of Groningen, Nijenborgh 4, 9747 AG Groningen, (The Netherlands); ‡ Leiden Institute of Chemistry, Leiden University, Einsteinweg 55, 2333 CC Leiden, The Netherlands

## Abstract

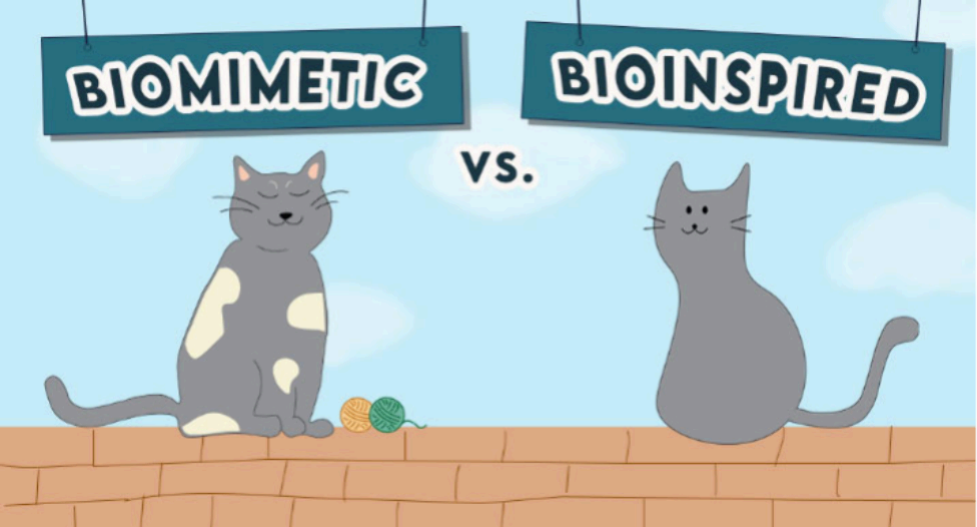

The terms biomimetic and bioinspired are very relevant in the field of bioinorganic chemistry and have been widely applied. Although they were defined by the International Organization for Standardization in 2015, these terms have at times been used rather ambiguously in the literature. This may be due to the inherent complexity of bioinorganic systems where, for example, a structural model of an enzyme active site may not replicate its function. Conversely, the function of an enzyme may be reproduced in a system where the structure does not resemble the enzyme’s active site. To address this, we suggest definitions for the terms biomimetic and bioinspired wherein structure and function have been decoupled. With the help of some representative case studies we have outlined the challenges that may arise and make suggestions on how to apply terminology with careful intention.

## Abstract 2

The terms biomimetic and bioinspired are relevant to the field of bioinorganic chemistry. Despite this, the two terms have been used rather ambiguously in the literature. In this viewpoint article, we propose definitions for the terms biomimetic and bioinspired wherein structure and function have been decoupled. With the help of some case studies, we discuss how to apply the terms to (complex) examples.

## Introduction

Nature’s unique optimization skills have led to the development of many extraordinary systems. Scientists have sought to both understand natural systems and use them as a toolbox for solving modern problems.^1^ Such drive has resulted in the introduction of new nomenclature to clarify the intention and level of replication of nature based approaches. Recently, many of these terms have been formally defined by the International Organization for Standardization (ISO).^2^ Out of these, two terms are particularly popular: biomimetics and bioinspiration. The ISO defines biomimetics as “interdisciplinary cooperation of biology and technology or other fields of innovation with the goal of solving practical problems through the function analysis of biological systems, their abstraction into models, and the transfer into and application of these models to the solution” and bioinspiration as “creative approach based on the observation of biological systems”.^2^ The interpretation and application of this terminology has further been discussed for science and engineering in general,^3,4^ and within specific subfields.^5,6^ Definitions for the terms biomimetic and bioinspired were briefly described for a specific application within bioinorganic chemistry by Lippard in 2006.^6^ However, despite their frequent usage, the terms biomimetic and bioinspired would benefit from a definition and in depth discussion specifically for their application within the broader context of the field of bioinorganic chemistry.

The terms biomimetic and bioinspired have been used somewhat interchangeably,^3,5^ and even loosely in an opportunistic fashion, in the field of bioinorganic chemistry. This may be a result of the broad scope that the field encompasses, which ranges from studying the (natural) reactivity of (engineered) metalloenzymes (such as cytochrome P450s),^7−10^ to transient metal complexes with similar reactivities to metalloenzymes (including high valent oxoiron complexes),^11−14^ to simple metal complexes that are not biomimetic or bioinspired but are designed to interact with biological systems (*cis*-platin being an important example).^15^ Such diversity in perspectives can lead to different intentions for similar systems, eventually making it difficult to distinguish whether a biological system is being replicated or merely serving as inspiration. The relaxed usage of the terms “biomimetic” and “bioinspired” within bioinorganic chemistry may also stem from the particularly complex relationship between structure and function for bioinorganic systems,^16^ which renders adequate application of these terms rather subtle.

## Definitions

**Figure 1:**
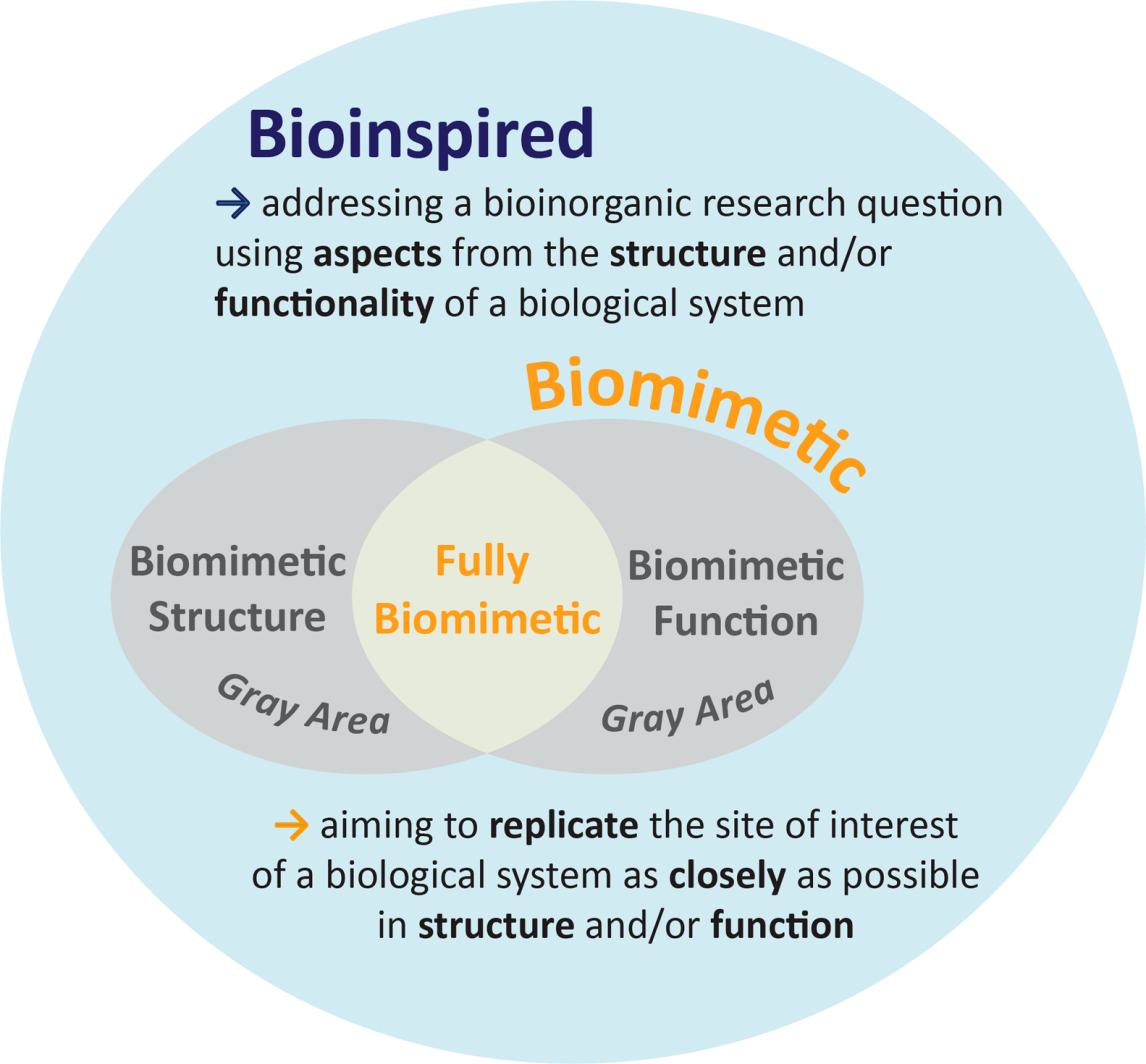
Diagram showing the relationship between the terms bioinspired and biomimetic and their definitions proposed in this work.

To provide a systematic framework, we propose the following definition for biomimetic bioinorganic chemistry: “aiming to replicate the site of interest of a biological system as closely as possible in structure and/or function”; and the following definition for bioinspired bioinorganic chemistry: “addressing a bioinorganic research question using aspects from the structure and/or functionality of a biological system”. In our definitions, we have consciously decoupled structure and function, allowing for a system to have a biomimetic structure and a bioinspired function, or *vice versa*. It is only when both structure and function are considered to be biomimetic that we would consider the system as a whole to be biomimetic. In cases where only the structure is biomimetic, the system can still be considered biomimetic in structure. However, we may consider the system as a whole to be in a somewhat gray area and a user is advised to make a conscious choice in their wording. Overall, biomimetic can be considered a subcategory of bioinspiration as it is more restrictive in how closely the system must resemble the model biological system (Figure 1).

Determining when it is appropriate to call a system biomimetic or bioinspired can be somewhat challenging in certain cases. A perfectly biomimetic system, where all aspects of the biological system are replicated, is generally unachievable and often unnecessary. Although every case is different, we suggest that as a general guideline a structure be called biomimetic once the first coordination sphere, the important elements of the second coordination sphere, and spin state of the biological system have been replicated. Similarly, we suggest that a system can be considered to have a biomimetic function when the overall reactivity, mechanism, and selectivity of the biological system are replicated. Situations may occur where, for the specific focus of the study, it is only relevant to replicate the structure or function up to a certain point. In these cases, authors may choose to indicate the scope within which they are considering their system to be biomimetic.

It is further important to acknowledge that the intention of a study can influence the terminology that an author may choose to apply. In certain instances, the focus of a study may encompass the development of a model system which is used to gain further understanding of the biological system. In other cases, aspects of the biological system may be replicated to develop a methodology with societal relevance. This distinction in perspectives can have an influence on the relevant nomenclature. There where the classification between bioinspired and biomimetic is not straightforward authors are encouraged to clarify their classification in the context of their scope and intention, research question or advances reported in their study.

## Case Studies

Within this viewpoint, three representative case studies are used to exemplify the diversity of structure–function considerations that are relevant within the field of bioinorganic chemistry and the resulting complexity in the application of the terms biomimetic and bioinspired. Research based on the chlorination reactivity of chloroperoxidase will be used to exemplify a relatively straightforward example wherein biomimetic structures lead to biomimetic reactivity, and bioinspired structures lead to bioinspired reactivity (Case 1). Nitrite reduction inspired by nitrite reductase will be used to outline the importance of second coordination sphere interactions in replicating enzymatic function in a biomimetic way (Case 2). Lastly, the contrasting reactivity of cytochrome P450 and superoxide reductase will be highlighted and compared to that of nonheme iron complexes to show how in certain cases function is dictated mainly by the spin state of the metal center rather than (coordination environment) structure models of the enzyme (Case 3). We would like to emphasize that these examples are not an exhaustive overview of the field, but are selected to demonstrate certain important features relevant to the definitions presented.

### Chloroperoxidase: Clear Biomimetic and Bioinspired Chemistry (Case 1)

**Figure 2:**
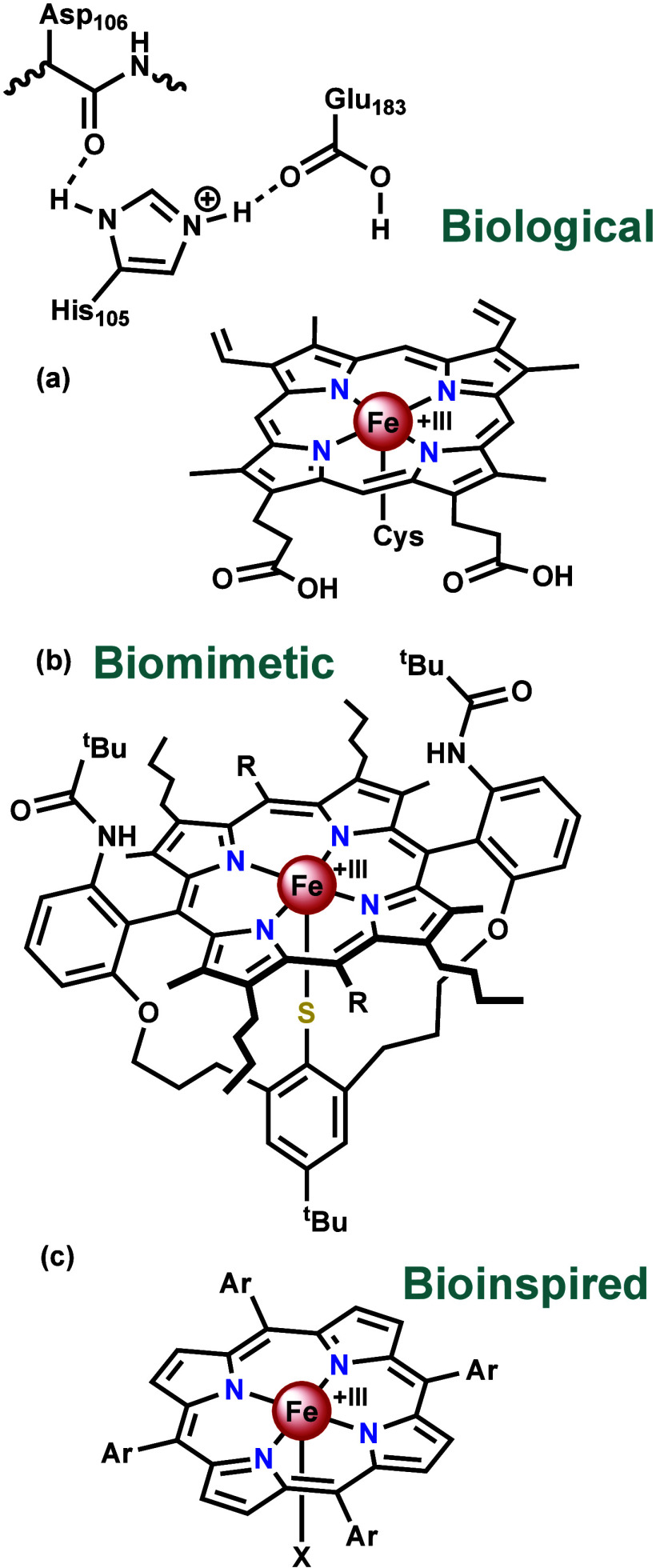
Structures of (**a**) the active site of chloroperoxidase,^19,20^ (**b**) active site models developed by the Woggon group, where R = H or C_6_F_5_,^32,33^ (**c**) simple, meso-substituted porphyrins used for stoichiometric electrophilic chlorinations by the Fujii group, X = NO_3_^–^, and Ar = pentafluorophenyl,^34^ and the catalytic umpolung of chloride by the Klein group, where X = ^–^OC(O)CF_3_, and Ar = phenyl, or 2,6-difluorophenyl.^35^

**Scheme 1:**
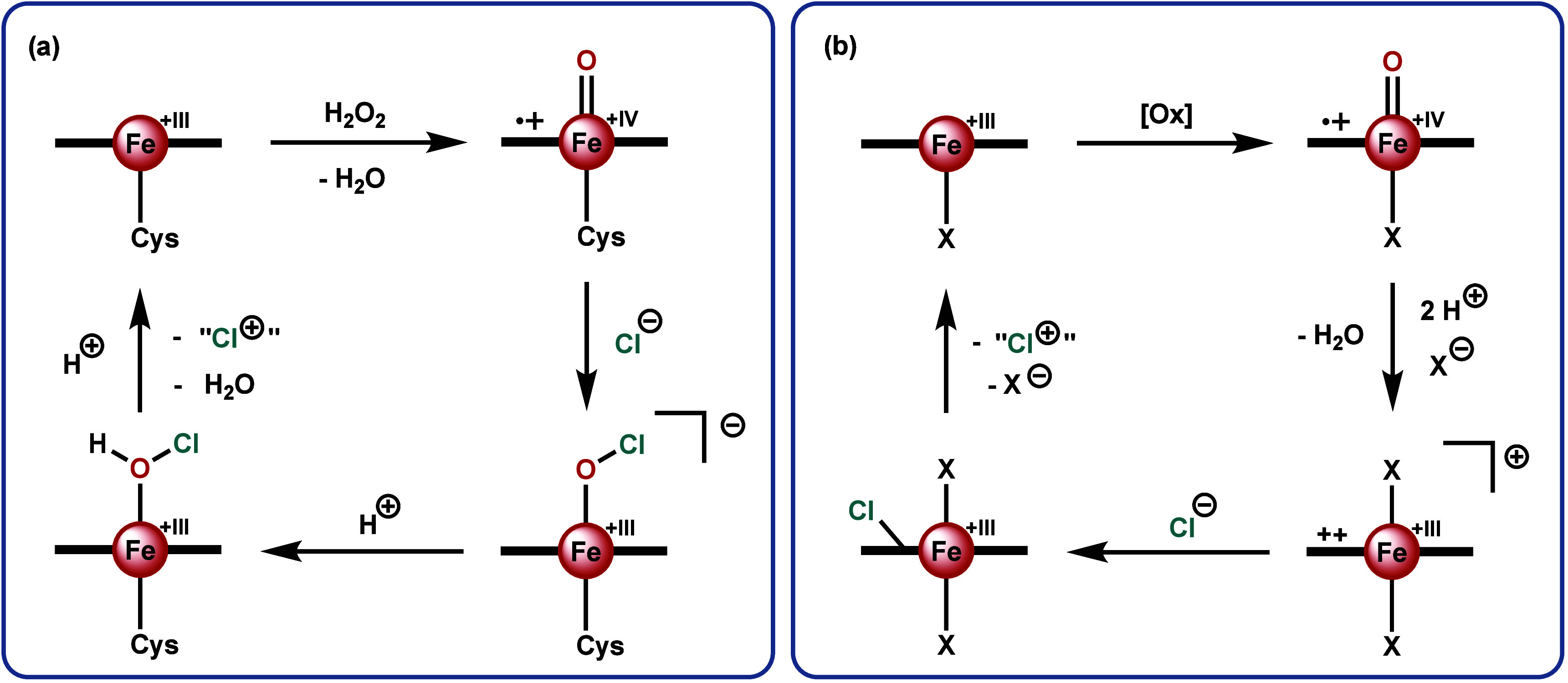
Mechanisms for the Electrophilic Chlorination Reactivity by (a) Chloroperoxidase and (b) Aryl *meso*-Substituted Porphyrins The equatorial ligand, depicted as a horizontal bold line, is protoporphyrin IX in (**a**) and a *meso*-substituted porphyrin in (**b**). [Ox] refers to any oxidant capable of oxidizing Fe(III)-porphyrin to Compound I. X represents any anionic coordinating ligand in solution. Reproduced with adaptation from ref (36). Copyright 2022 American Chemical Society.

Chloroperoxidase is a monomeric, heme-containing enzyme with a protoporphyrin IX equatorial ligand and cysteine axial ligand (a feature generally reserved for cytochrome P450s), see Figure 2a.^17−20^ In the resting state, the iron center is in the + III oxidation state.^21^ Second coordination sphere interactions include polar residues on the distal side of the heme.^19,20^ These are thought to be involved in peroxide binding and speculated to interact with a hypochlorite intermediate.^20^ Although chloroperoxidase is capable of performing a wide variety of reactivities common to peroxidases, catalases, and cytochrome P450s,^22^ its oxidative chlorination reactivity is rather unique and fascinating.^23−25^ After oxidation by hydrogen peroxide (H_2_O_2_) to Compound I (the general term for an oxoiron(IV) with a singly oxidized porphyrin ring),^26,27^ chloride attacks the oxido ligand to generate an iron(III) hypochlorite species (Scheme 1a).^21,28−30^ Herein the chlorine is electropositive, allowing it to be transferred as a “Cl^+^” and electrophilically chlorinate a substrate. It is likely that protonation of the hypochlorite occurs prior to the chlorination step.^20,31^

Soon after the X-ray crystal structure of chloroperoxidase was solved,^19^ the Woggon group reported the synthesis of chloroperoxidase active site models (Figure 2b).^32,33^ These were specifically developed with the intention to mimic enzymatic activity in attempts to further understand the biological reaction mechanism.^32^ The catalyst design took into consideration not only the porphyrin ring, but also the thiolate axial ligand and proton binding sites which had been identified in the crystal structure. These complexes replicate the resting state of the enzyme active site as closely as reasonably attainable up to the second coordination sphere and we therefore consider the term biomimetic to be justified here in relation to its structure.

Upon reaction of these active site model complexes with benzyltriethylammonium hypochlorite, the iron(III) hypochlorite (FeOCl) complex was generated.^32,33^ This same species could be generated by oxidation of the iron(III) complex with H_2_O_2_ to form Compound I and subsequently adding benzyltriethylammonium chloride.^32^ The iron(III) hypochlorite showed very little chlorination reactivity of monochloro-dimedone, a commonly used substrate for chloroperoxidase. However, addition of acetic acid yielded iron-bound HOCl (FeO(H)Cl), which is active for the chlorination of monochloro-dimedone.^32^ This reactivity was also shown to work under catalytic conditions, with turnover numbers ranging from 37 to 420.^33^

It should be noted that, at the time, the enzymatic chlorination reactivity of chloroperoxidase was not fully understood. In particular, there was no evidence yet of either the Fe-OCl or Fe–O(H)Cl adducts. It is the discovery of these adducts in the biomimetic complexes and the understanding of their spectroscopic characteristics that allowed the FeOCl and FeO(H)Cl species to be observed in the enzyme active site.^31^ These active site models thus achieve their intended purpose and replicate the enzyme’s function. We therefore consider it appropriate to call these systems fully biomimetic.

Considering the complexity of the active site models developed by the Woggon group, there was an interest to reproduce this reactivity with simple aryl *meso*-substituted porphyrins (Figure 2c). These readily accessible complexes may be more suitable for industrial applications and possibly lead to an environmentally benign method for electrophilic chlorinations.^36^*meso*-Substituted porphyrins are similar to the active site of chloroperoxidase only in the coordination of iron(III) to a porphyrin ring. They differ in that the porphyrins are *meso*-substituted rather than β-substituted, they do not have a thiolate axial ligand, and there are no proton donor groups to mimic the polar residues on the distal side of the heme. We therefore consider the structure of these simple aryl *meso*-substituted porphyrins to be much less biomimetic than the Woggon complexes. In particular, considering that the intention of the use of *meso*-substituted porphyrins is to have a simple, readily accessible catalyst, we suggest that they be referred to as bioinspired in structure.

Addition of tetrabutylammonium hypochlorite to aryl *meso*-substituted porphyrins leads to the formation of a six-coordinate FeOCl or Fe(OCl)_2_ species depending on the conditions.^37,38^ However, these species decompose via heterolytic bond cleavage to generate Compound I and a chloride anion.^38^ This is the inverse reaction of that performed by chloroperoxidase, indicating that the thermodynamics of hypochlorite formation via a biomimetic pathway are unfavorable. Indeed, it was shown that addition of chloride to Compound I does not lead to hypochlorite formation.^38,39^ If chloride is added to Compound I in the presence of excess trifluoroacetic acid, mimicking the acidic conditions required for enzymatic activity, alternative reactivity is observed.^34^

The addition of trifluoroacetic acid to Compound I leads to the formation of an iron(III) π-dication species, wherein the ligand is neutral instead of doubly anionic (Scheme 1b).^35,40^ Nucleophiles, such as chloride, can attack the electron-poor porphyrin ring in order to form the corresponding isoporphyrin.^40^ The porphyrin-bound chloride can then be transferred to a substrate (e.g., trimethoxybenzene) as a “Cl^+^”, thereby leaving behind both electrons in the C–Cl bond and rearomatizing the porphyrin ring.^34^ Such reactivity has been shown to also work under catalytic conditions.^34,35^ Considering that the umpolung of chloride is achieved with bioinspired complexes, but the mechanism diverges from that for chloroperoxidase, we consider the chlorination function of aryl *meso*-substituted porphyrins to be bioinspired, rendering the whole system bioinspired.

According to our suggested definitions, chloroperoxidase has thus prompted the development of two different types of catalysis: biomimetic electrophilic chlorinations proceeding through an iron(III) hypochlorite, and bioinspired electrophilic chlorinations proceeding through an iron(III) *meso*-chloroisporphyrin. This exemplifies how designing complexes that are significantly simplified from the active site structure of an enzyme, and are thus synthetically more accessible, may render interesting bioinspired reactivities with the potential to solve problems of immediate societal relevance.

### Copper Nitrite Reductase: Challenges in Biomimetic Design (Case 2)

**Figure 3:**
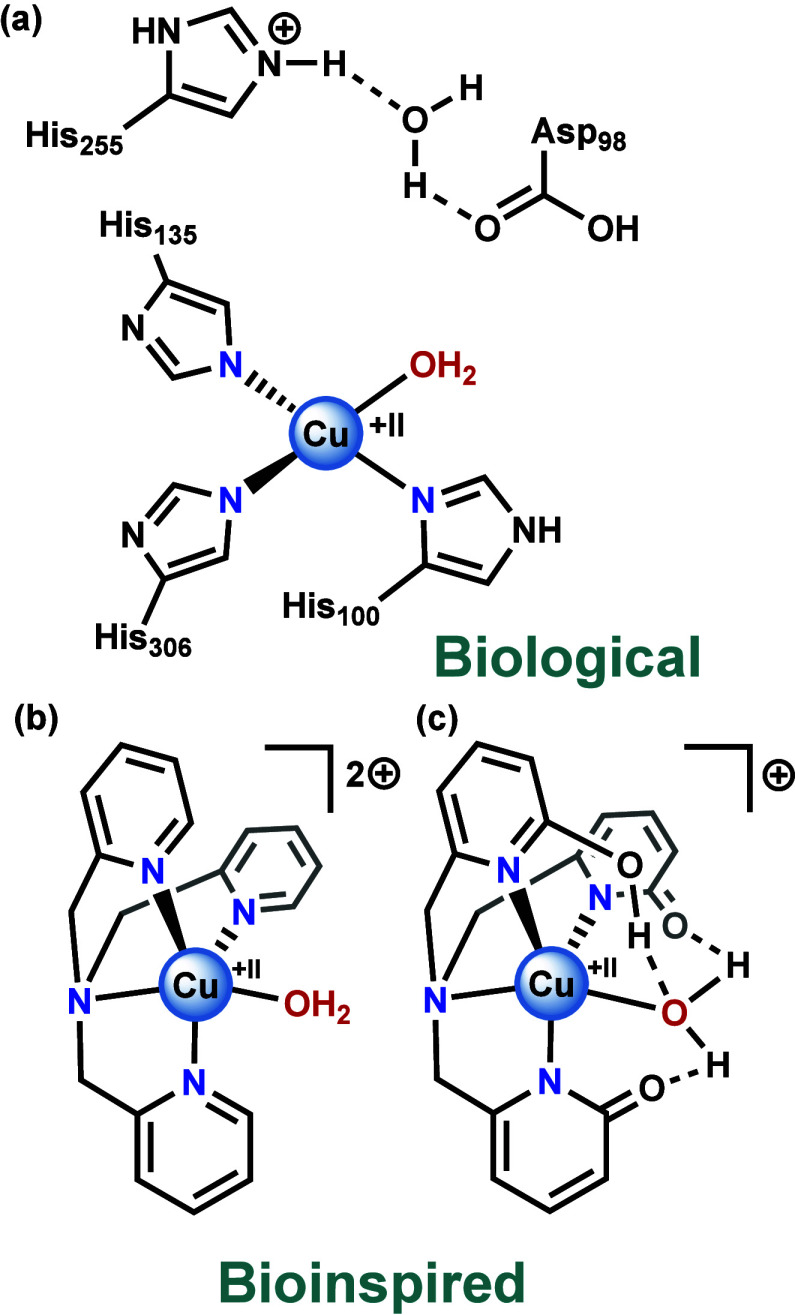
Structures of (**a**) the active site of copper nitrite reductase, (**b**) Cu(TPA), one of the many examples of an active site model of copper nitrite reductase, and (**c**) an active site model of copper nitrite reductase including a proton responsive ligand, as reported by the group of Szymczak.^53^

Copper nitrite reductase is a copper-based metalloenzyme that carries out the one-electron reduction of nitrite to nitric oxide, an important step in the process of denitrification (eq 1).^41^ Copper nitrite reductases contain two copper centers, a type 1 (T1) Cu site that is responsible for electron transfer,^42^ and a type 2 (T2) Cu center where nitrite coordination and reduction take place. In the resting state of the enzyme the T2 Cu active site is in the + II oxidation state and adopts a distorted tetrahedral geometry. Within the primary coordination sphere, three histidine amino acids and one water molecule can be found (Figure 3a). In addition, two amino acid residues located in the second coordination sphere, namely Asp98 and His255, are important for nitrite reduction activity by enabling proton transfer to the copper center.^43^
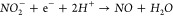
1

Although nitrite reduction is a relatively simple one-electron process, certain details of the mechanism of copper nitrite reductase are still being debated, with recent insights obtained from computational techniques contributing to this discussion.^44−48^ According to the first mechanisms proposed for copper nitrite reductase, nitrite binds to the oxidized copper center in a η^2^-O,O fashion, as is observed in multiple crystal structures.^49−52^ A hydrogen bond forms upon nitrite binding between the substrate and Asp98, resulting in elongation of one of the N–O bonds, which is ultimately important for proton transfer.^43,50^

**Scheme 2:**
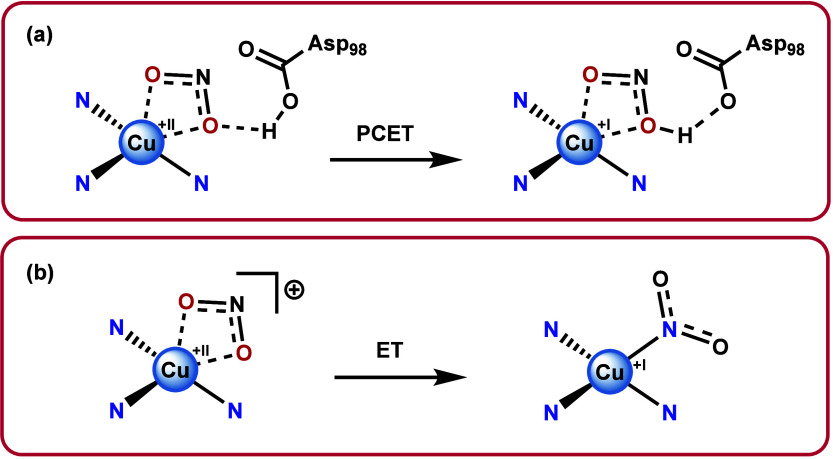
Mechanism of Electron Transfer and Binding Modes of Nitrite (**a**) Mechanism of proton-coupled electron transfer that takes place upon binding of nitrite by the active site of copper nitrite reductases. (**b**) Binding modes of nitrite found in copper nitrite reductases model complexes in both Cu(II) state and the Cu(I) after electron transfer. In both figures the coordination environment of the copper center is simplified for clarity.

In various works the electron and proton transfer steps following substrate binding were investigated. According to computational investigations, nitrite binding to Cu(II) induces protonation of the Asp98 site, thereby raising the redox potential of the T2 site^44^ and triggering electron transfer from the T1 to the T2 site. In line with this, lowering of the pH will trigger electron transfer. Specifically the proton transfer from Asp98 to Cu(II)-NO_2_ is required for electron transfer to the T2 site.^45^ More recently it has been established that these proton and electron transfer steps are coupled,^54,55^ and could be modeled by QM/MM MD (Scheme 2a).^47^ In earlier work it was proposed that after electron and proton transfer a Cu(II)–OH_2_ or Cu(II)–OH species would form, leading to release of a NO molecule.^51^ This is in line with several later computational works^45,47,48^ and a recent high-resolution neutron crystallography study which confirmed the presence of a Cu(II)–OH species in the resting state of the enzyme.^56^

A few years before the first mechanisms on copper nitrite reductases were reported, the first active site models of the enzyme were developed.^57−63^ In the following years, a wide variety of ligands were developed to generate numerous model compounds. In general, these complexes involve the interaction between a single copper site and a tridentate or tetradentate ligand with N-coordinating groups, like pyridine or imidazole, reminiscent of the histidine residues present in the T2 Cu site.^52^ Of these model complexes, Cu(TPA) ([Cu(TPA)(H_2_O)]^2+^, TPA = tris(2-pyridylmethyl)amine), containing a tripodal tetradentate ligand (Figure 3b), is a well-studied complex for nitrite reduction.^64−68^ Notably, these mononuclear copper complexes with N-coordinating ligands were designed to be bioinspired in structure, as the first coordination sphere is not fully replicated and the second coordination sphere is not featured.

In terms of reactivity, various studies have demonstrated that these model complexes are able to produce NO upon addition of two equivalents of acid to a Cu(I) species^59,60,69−78^ or by electrochemical means.^64−67,79−86^ In line with the enzymatic pathway, the η_2_-O,O binding mode predominates in the majority of reported crystal structures of Cu(II) complexes.^58,61,69,72,73,76,79,80^ However, there is no evidence that copper binds nitrite in a O-bound fashion upon reduction, as only crystal structures featuring N-bound Cu(I)-NO_2_ species exist.^59,60,69,70,72−74,79,80^ This has led to general consensus that the nitrite reduction mechanism carried out by model complexes takes place via the N-bound pathway (Scheme 2b).

Remarkably, only a few studies have investigated the exact order of the electron and proton transfer steps within the bioinspired pathway. A recent study by one of our groups, using Cu(TPA) as an active site model, demonstrated that electron transfer is succeeded by two proton transfer steps. Moreover, it was shown that the electrocatalytic nitrite reduction mechanism in aqueous solution takes place via a general acid-catalyzed mechanism.^66^ This observation indicates that proton delivery in the bioinspired pathway is strongly dependent on the proton source, in line with the enzymatic activity. In our opinion, due to the overall mechanistic divergence, these simplified model compounds are best described as bioinspired in both their structure and reactivity, although they are in general active catalysts for the reduction of nitrite.

As to date, only four works have been reported in which the ligand structure of copper nitrite reductase model complexes was modified with the aim to create compounds that resemble the enzyme active site in more detail.^53,87−89^ In all cases a hydrogen-bonding moiety was incorporated in the ligand to resemble the enzymatic second coordination sphere including the Asp98 and His255 amino acids. Regarding their reactivity, DFT calculations on a copper complex with a tetradentate tripodal ligand featuring a carboxylic acid moiety showed that this complex can deliver protons to the nitrite bound substrate via its acid group via a proton-coupled electron transfer (PCET) step.^87^ In a similar way, catalysts with tridentate ligands that incorporate different hydrogen-bonding moieties are likely to carry out the nitrite reduction reaction via a proton-donating pathway on the ligand.^88^ The reduction of nitrite at a copper complex with a proton-responsive cryptate ligand resulted in the release of NO from NO_2_^–^ in a phenol-mediated pathway when a proton in the outer coordination sphere was absent.^89^ However, in the presence of this proton, anaerobic phenol oxidation was facilitated.^89^ Interestingly, DFT calculations showed that a modified TPA-like ligand that incorporated three proton responsive OH-moieties (Figure 3c) enabled the Cu(I) center to bind nitrite in a η^1^-κO fashion, contrasting the commonly observed N-bound species.^53^ This suggests that the hydrogen bonding network of this ligand closely resembles the H-bonding network of the amino acids in the second coordination sphere of enzymes, leading to a more biomimetic structure.

Next to the design of model complexes, we want to point out that *de novo* metalloproteins provide an alternative platform for the development of nitrite reductase model systems.^90,91^ In this approach the interaction between a protein scaffold and a metal site are investigated by tuning main structural features in order to understand protein function. Studies on such *de novo*-designed peptides for nitrite reductase, which incorporate a T2 copper site into the interior of three-stranded coiled coils, have shown that modulations in the peptide structure will alter the catalytic activity, redox potential, and binding affinities of the system.^92^ In addition, modification of the second coordination sphere interactions affect the metal coordination environment, resulting in an increase of nitrite reduction activity up to 75 times.^93^ Furthermore, by incorporation of the T2 site into a different, asymmetric protein matrix, the catalytic activity could be further increased.^90^ Compared to native copper nitrite reductase, the activity of *de novo*-designed protein systems is still 3 to 4 orders of magnitude lower, likely because the second coordination sphere interactions as present in the native enzyme could not be fully incorporated yet.^91,94^ In terms of structure, we therefore consider these *de novo* metalloproteins to be bioinspired, although they are more biomimetic than the model metal complexes that have been developed thus far. In terms of function, it has not been reported if O-bound nitrite is formed upon reduction of the copper site, and therefore the function of the systems cannot be considered biomimetic thus far.

Taken together, the development of copper compounds to resemble copper nitrite reductase activity has resulted in the availability of a wide range of structural models and *de novo* metalloproteins that are active for (electro)catalytic nitrite reduction. The molecular models primarily differ from the enzymatic pathway in terms of the nitrite binding mode, as the N-bound pathway is considered to be their mode of action during catalysis. More recently, active site models incorporating ligands with proton-responsive groups have been developed, suggesting that this will alter the nitrite reduction mechanism. As the proton responsive ligands are able to deliver protons to the copper-bound substrates, we would recognize that their structures have some biomimetic aspects. Considering that the second coordination sphere still does not resemble that of the enzyme, we would overall consider this compound to be bioinspired. Regarding *de novo*-designed protein systems, the second coordination sphere could not be fully modeled, resulting in a lower activity than that of the native enzyme.^91^ We anticipate that one might be tempted to consider active site models incorporating ligands with proton-responsive groups or *de novo* metalloproteins to be biomimetic in their activity. However, whether incorporation of a proton-responsive group in copper-based model compounds or *de novo* enzyme design will result in the O-bound mechanism, as observed in the enzymatic pathway, is only speculated based on a single DFT study to date in case of model compunds^53^ and not been reported for the *de novo* enzyme systems. Therefore, despite ongoing efforts, copper nitrite reductases provide an example of a bioinorganic system for which no fully biomimetic active site models have been published yet. Future studies should aim at designing ligands that allow for a distorted tetrahedral geometry and better replication of the second coordination sphere that will stir the mechanism to take place via the O-bound pathway.

### Cytochrome P450 and Superoxide Reductase: The Added Complexity of Spin States (Case 3)

**Scheme 3:**
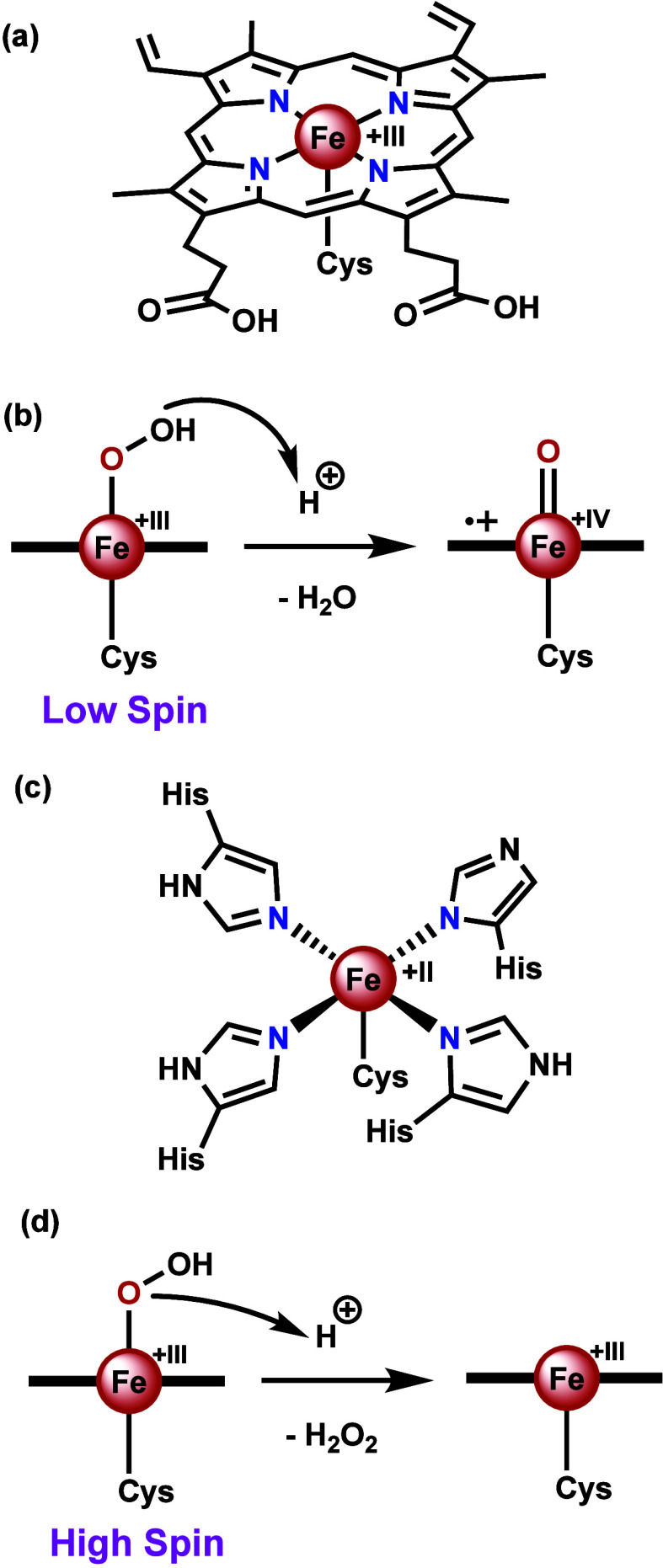
Active Site Coordination and Reactivity of Hydroperoxo of Cytochrome P450 and Superoxide Reductase (**a**) Active site coordination of cytochrome P450, (**b**) reactivity of the hydroperoxo in cytochrome P450 towards protonation, (**c**) active site coordination of superoxide reductase, (**d**) reactivity of the hydroperoxo in superoxide reductase towards protonation. The equatorial (nitrogen donating) ligand(s) are depicted as a horizontal bold line in (**c**) and (**d**).

Cytochrome P450 and superoxide reductase are two enzymes which share the same heteroatoms in the same coordination sphere. They have a similar geometry in their active site, but have vastly different reactivities and functions. Cytochrome P450 is a heme containing monooxygenase, ligated to the enzyme via a cysteine residue (Scheme 3a, similar to chloroperoxidase, *vide supra*).^95,96^ It is capable of reducing dioxygen (O_2_) to water, forming Compound I in the process, which can in turn oxidize a wide variety of substrates.^8,97^ Superoxide reductase is an iron-containing enzyme in which the metal center is coordinated to four histidines in a planar fashion and one cysteine axially (Scheme 3c).^98,99^ Superoxide reductase thus has the same atoms coordinating in a very similar geometry compared to cytochrome P450.^100^ In contrast to cytochrome P450, superoxide reductase will reduce the harmful superoxide anion (O_2_^–·^) to H_2_O_2_.^98,99^

Both cytochrome P450 and superoxide reductase generate an iron(III) hydroperoxo (FeOOH) species. Although both FeOOH species are coordinated to four nitrogens equatorially, the ligand fields that arise from these nitrogens are different, leading to different spin ground states. For cytochrome P450 the FeOOH species has a low spin iron center, whereas for superoxide reductase it is high spin. It has been proposed that spin state is one of the main factors that influences the reactivity of the FeOOH species.^101^ For a low spin FeOOH, the O–O bond is weakened relative to the Fe–O.^101^ This leads to protonation at the distal oxygen, causing release of water and generating Compound I upon heterolytic cleavage of the O–O bond (Scheme 3b).^101^ When the metal center is high spin, the Fe–O bond is weakened relative to the O–O bond, and protonation occurs at the proximal oxygen, leading to dissociation of H_2_O_2_ (Scheme 3d).^101^

**Figure 4:**
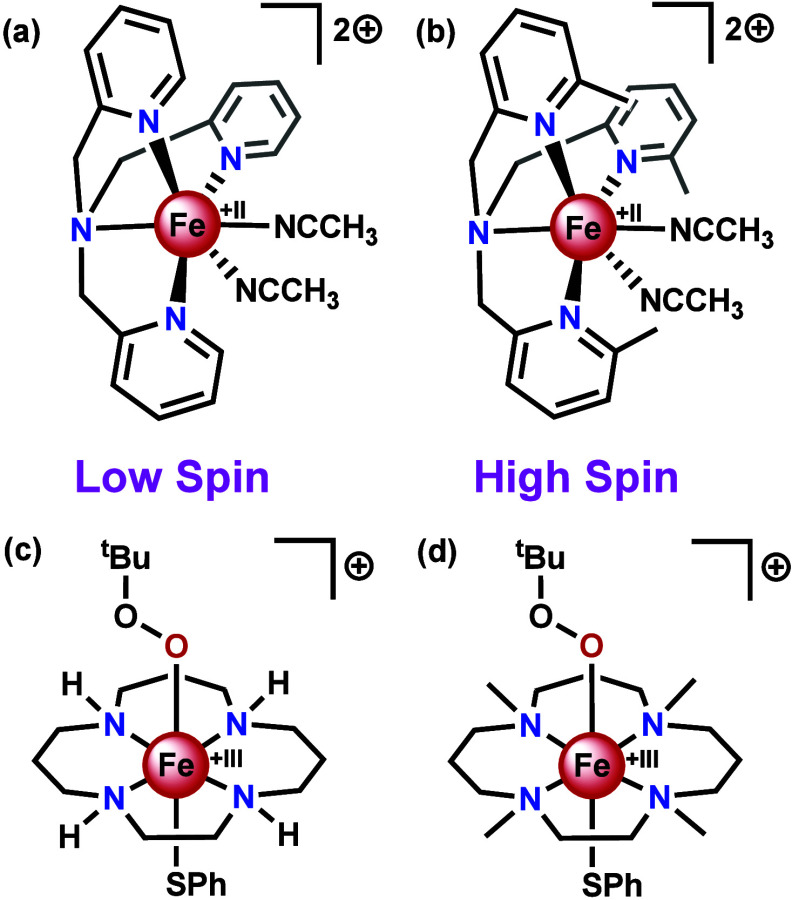
Structures of (**a**) [Fe(TPA)(CH_3_CN)_2_]^2+^, (**b**) [Fe(6-Me_3_TPA)(CH_3_CN)_2_]^2+^, (**c**) [Fe([15]aneN_4_)(SPh)(OO^t^Bu)]^+^, (**d**) [Fe(Me_4_[15]aneN_4_)(SPh)(OO^t^Bu)]^+^.

In order to generate compounds which can replicate the reactivities of cytochrome P450 or superoxide reductase, the spin state of the metal complex must be appropriate. Controlling spin states can be rather challenging, as small ligand modifications can significantly alter the spin state. An example is the methylation of the TPA ligand at the 6-position (6-Me_3_TPA). Whereas [Fe(TPA)(CH_3_CN)_2_](ClO_4_)_2_ (Figure 4a) has a low spin metal center, [Fe(6-Me_3_TPA)(CH_3_CN)_2_](ClO_4_)_2_ (Figure 4b) is a high spin complex.^102^

For both of these compounds, Fe(III)OO^t^Bu species have been generated by reaction with *tert*-butyl hydroperoxide (^t^BuOOH) and the spin-state of the parent compound is conserved.^102^ The Fe-OO and FeO-O bond strengths can conveniently be analyzed by resonance Raman spectroscopy. Similar to the enzymatic cases, it was found that the FeO-O bond is weaker for the low spin compound than the high spin compound, and the Fe-OO bond is weaker for the high spin compound than the low spin compound.^102,103^ Reaction of [Fe(TPA)(CH_3_CN)_2_]^2+^ with H_2_O_2_ is known to equally form an Fe(III)OOH species with a low spin iron center.^104^ Furthermore, [Fe(TPA)(CH_3_CN)_2_]^2+^ has been shown to be an active catalyst for hydroxylations using H_2_O_2_ under acidic conditions.^104,105^ Mechanistic studies suggest that protonation of the Fe(III)OOH occurs at the distal oxygen, leading to O–O bond cleavage and formation of an high valent oxoiron compound.^105−108^ Despite the very different coordination environment compared to cytochrome P450, it exhibits related reactivity for cytochrome P450 that might be viewed as biomimetic in function.

A compound that somewhat more closely resembles the coordination environment of cytochrome P450 and superoxide reductase than TPA is [Fe([15]aneN_4_)(SPh)]BF_4_, where [15]aneN_4_ = (1,4,8,12-tetraazacyclo-pentadecane). Similarly to the enzymes, this compound has four nitrogen donor atoms that occupy the equatorial positions and a thiolate as axial ligand.^109^ Although this compound has a high spin iron center, it generates a low spin FeOO^t^Bu species (Figure 4c) upon reaction with ^t^BuOOH.^109^ [Fe([15]aneN_4_)(SPh)]BF_4_ can thus be considered biomimetic in structure until the first coordination sphere and including spin state. A striking feature of this FeOO^t^Bu species is that, beyond its expected weak FeO-O bond, it has a particularly weak Fe-OO bond compared to other low spin alkyl peroxo species, which was attributed to the presence the thiolate ligand *trans* to it.^109,110^ A similar compound, [Fe(Me_4_[15]aneN_4_)(SPh)]BPh_4_, where the four nitrogen atoms have been methylated, reacts with ^t^BuOOH to generate a high spin FeOO^t^Bu species (Figure 4d).^111^ Again, the Fe-OO bond was found to be particularly weak compared to other high spin alkyl peroxo species.^111^ The analogous compound with a triflate *trans* to the alkyl peroxo rather than a thiolate was found to have a very similar FeO-O Raman shift, but a stronger Fe-OO bond, with a Raman shift more comparable to other high spin alkyl peroxo species.^111^ Hence, beyond spin state, the *trans* effect is a further aspect to consider. [Fe(Me_4_[15]aneN_4_)(SPh)(OO^t^Bu)]^+^ was also found to react with acid to release ^t^BuOOH,^112^ thereby mimicking the reactivity of superoxide reductase.

These examples illustrate the complexity in tuning reactivity in a bioinorganic context. Spin state is an intrinsic feature of bioinorganic chemistry and may be considered to be a key structural feature. Simply replicating the appropriate coordination geometry and the same donor atoms may not lead to the biomimetic reactivity profile if the spin state is not considered. Spin state is thus highly relevant to take into consideration within the scope of assigning the terms biomimetic and bioinspired.

## Conclusion

Although they are quite different, the terms biomimetic and bioinspired have at times been used rather ambiguously in the literature. To aid the deliberate use of these terms we have proposed definitions which decouple structure and function. In this manner a structure can be considered biomimetic despite the enzymatic function not being replicated and *vice versa*. However, we suggest that only when both structure and function are biomimetic that the entire system can be called biomimetic. In the case where only one of the two is considered biomimetic, the system as a whole is not so clearly defined. In these cases we suggest that authors carefully choose their phrasing. Placing the chosen term into context by describing the scope and intention of the research will aid clear communication. Although this may sound straightforward, there are many examples in which it may not be clear whether a structure or function should be considered biomimetic. With the help of case studies, we have outlined these complexities and hope to have encouraged careful application of the terms.
